# Advances in lung ultrasound

**DOI:** 10.1590/S1679-45082016MD3557

**Published:** 2016

**Authors:** Miguel José Francisco, Antonio Rahal, Fabio Augusto Cardillo Vieira, Paulo Savoia Dias da Silva, Marcelo Buarque de Gusmão Funari

**Affiliations:** 1 Hospital Israelita Albert Einstein, São Paulo, SP, Brazil

**Keywords:** Thorax/ultrasonography, Pleura, Pleural effusion

## Abstract

Ultrasound examination of the chest has advanced in recent decades. This imaging modality is currently used to diagnose several pathological conditions and provides qualitative and quantitative information. Acoustic barriers represented by the aerated lungs and the bony framework of the chest generate well-described sonographic artifacts that can be used as diagnostic aids. The normal pleural line and A, B, C, E and Z lines (also known as false B lines) are artifacts with specific characteristics. Lung consolidation and pneumothorax sonographic patterns are also well established. Some scanning protocols have been used in patient management. The Blue, FALLS and C.A.U.S.E. protocols are examples of algorithms using artifact combinations to achieve accurate diagnoses. Combined chest ultrasonography and radiography are often sufficient to diagnose and manage lung and chest wall conditions. Chest ultrasonography is a highly valuable diagnostic tool for radiologists, emergency and intensive care physicians.

## INTRODUCTION

Ultrasound examination of the chest has seen consistent advances over the last few decades, with current applications reaching far beyond pleural effusion investigation.

Developments in ultrasonography resulted not only in improved image acquisition and processing, but also in greater portability, with the advent of new, small-sized portable machines providing high image quality.

Thoracic and lung ultrasound stands out in this novel scenario, particularly in emergency and intensive care unit settings. Sonographic assessment of chest wall changes, including scanning of costal arches, pleural spaces and lung parenchyma can provide accurate qualitative and quantitative data.^(1,2)^

The major chest ultrasound applications are listed in this article. The imaging method has sensitivity and specificity indices ranging from 81 to 97% and 95 to 100%, respectively.^(3)^

The ability to recognize major sonographic findings associated with different diseases and clinical emergency conditions is important for radiologists, particularly those specializing in emergency and intensive care medicine. Pulmonologists, emergency medicine physicians and thoracic surgeons may also benefit from this knowledge, given its potential applicability in management of traumatic conditions and in intraoperative situations.^(3,4)^

This article provides a detailed description of chest ultrasonography. Major sonographic findings are described in a didactic manner. The applicability of the technique as a useful and accurate diagnostic tool in different medical specialties is emphasized.

## ULTRASOUND PHYSICS AND ANATOMY

The aerated lungs and the bony framework of the thorax are important barriers to sonographic examination. However, solid knowledge of related acoustic phenomena and artifacts can aid in sonographic diagnosis. As an example, and comparing to transabdominal ultrasonography, the artifacts such as posterior acoustic shadowing (suggestive of biliary and renal calculi) are widely used as diagnostic aids.

Subpleural acoustic artifacts once seen as limiting factors in sonographic assessment of the lungs and chest wall have been systematically described. Updated concepts have led to wider application of ultrasonography to the diagnosis of thoracic conditions that fell outside the scope of the technique in the past. Sonographic acoustic artifacts appear as lines and were given specific definitions in international standards. These lines can be used to investigate several conditions affecting the lung parenchyma and are described as follows:

### Echogram pattern of the normal pleura

**Figure 1:**
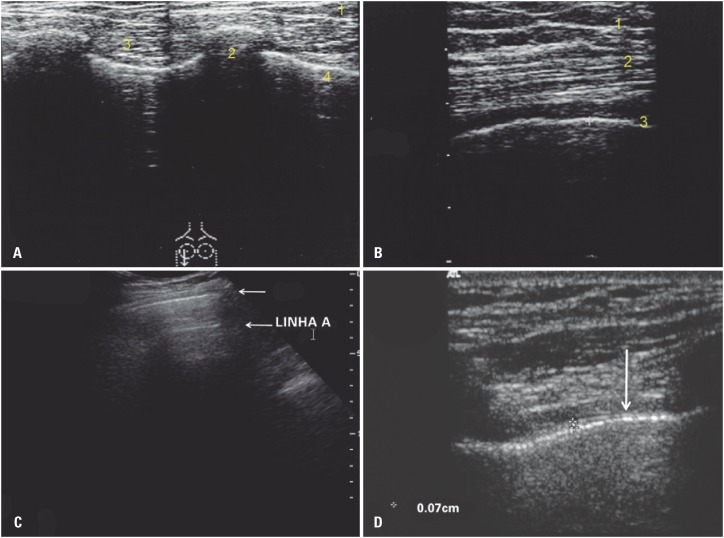
Sonographic anatomy. (A) Chest wall sonogram (7 MHz linear probe). (1) Observe the different tissue planes displayed: skin and subcutaneous tissue, (2) ribs (transverse plane), (3) intercostal muscles, (4) pleural line. (B) Sonographic anatomy of the chest wall (7 MHz linear probe). (1) Observe the different tissue planes displayed (from the surface down): skin and subcutaneous tissue, (2) pectoral muscles note the typical fibrillar appearance and the, (3) pleural line. (C) Echogram pattern of the normal pleura (3.5 MHz convex probe). Only the pleural line can be identified at this frequency; the parallel subpleural A line is enhanced (reverberation artifact). Low image resolution interferes with recognition of pleural line layers. (D). Pleural line. Both portions of the pleura (echoic lines indicated by measuring devices) and the anechoic space between them are displayed. The visceral and parietal pleuras correspond to the inner and outer echoic lines, respectively; the extrapleural hypoechoic line (arrow) corresponds to extrapleural fat. Note big image resolution differences; higher image resolution achieved with higher frequency probes enables detailed visualization of pleural line components

The echo produced by the pleura under normal conditions appears as a hyperechoic line gently and synchronously sliding with respiratory movements (Figure 1). This line and related artifacts serve as landmarks for sonographic diagnosis of thoracic conditions.

The chest wall is preferably scanned using a low frequency (3 to 5MHz) convex probe. All intercostal spaces are scanned; the patient is repositioned in bed for examination of the dorsum.

Pleural line components can be separated into visceral pleura, pleural space and parietal pleura using high-resolution probes (5 to 17MHz); evaluation of normal extrapleural fat is also possible. Thorough scanning takes between 10 and 15 minutes to complete.

### A Lines

**Figure 2:**
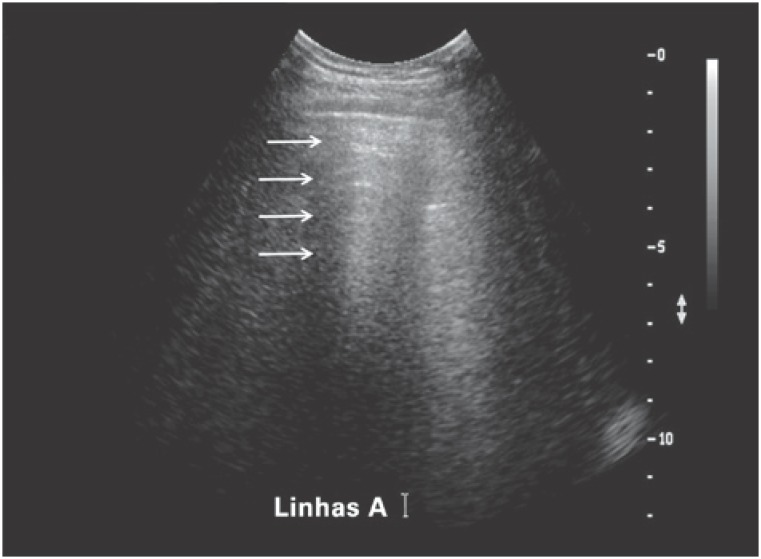
A lines represent reverberation artifacts of the pleuropulmonary interface. These lines are equidistant from each other and become less echoic with increasing depth (arrows)

A lines represent reverberation artifacts and appear as horizontal, parallel lines equidistant from each. These lines are commonly seen in healthy individuals (Figure 2) and may be erased (by B lines) or enhanced (in the presence of pneumothorax).^(3)^

### B Lines

**Figure 3:**
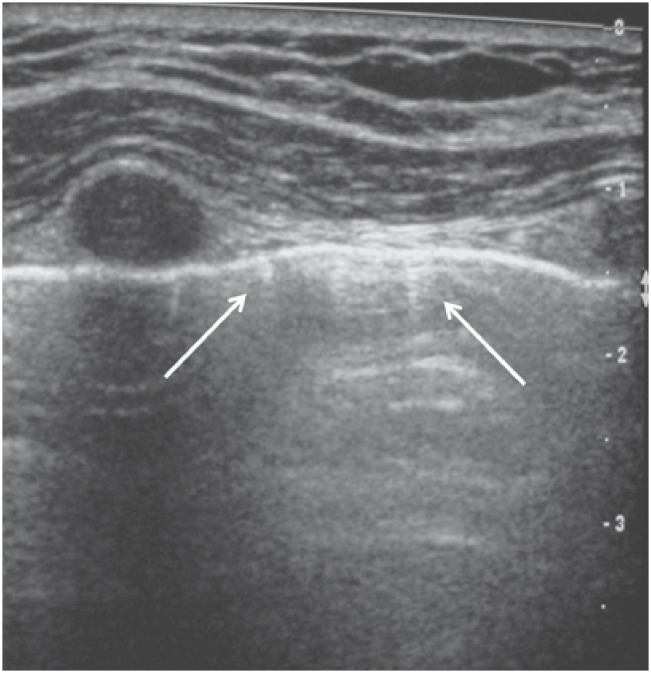
B lines appear as comet-tail artifacts (arrows) perpendicular to the pleural surface. These lines represent the reflecting interface between interlobular septa and the normal lung

B lines represent interlobular septa and appear as small, well-defined vertical comet-tail artifacts perpendicular to and arising from the pleural line. These lines move with the pleural line during respiration and may erase A lines (Figure 3). One or two of these lines may be seen per intercostal space in 30% of healthy individuals, particularly in dependent portions of the lung.

B lines indicate filling of intralobular or interlobular septa and are often seen in pulmonary edema and interstitial lung diseases.^(1,3)^

Thickened B lines may fuse together to form coalescent B lines representing peripheral lung ground glass opacities seen in high resolution computed tomography (CT).^(1,3)^

### C Lines

**Figure 4:**
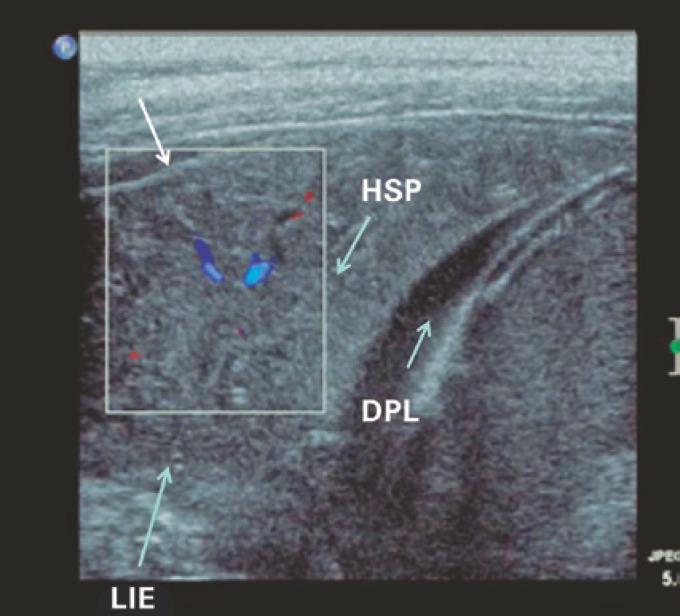
C lines (contained within the square). C lines are defined as hypoechoic subpleural focal images (generated by condensed lung tissue) without visceral pleural line gap (arrow) HSP: subpleural hypoechogenicities; DPL: pleural effusion; LIE: left lower lobe.

C lines are defined as hypoechoic subpleural focal images generated by condensed lung tissue, without visceral pleural line gap (Figure 4). C lines are not true lines, but are designated as such for the sake of consistency with nomenclature standards.

### False B lines (E and Z lines)

False B lines (E and Z lines) are vertical to the pleural line and may be mistaken for true B lines. However such lines represent different entities, as follows:

#### E lines

**Figure 5:**
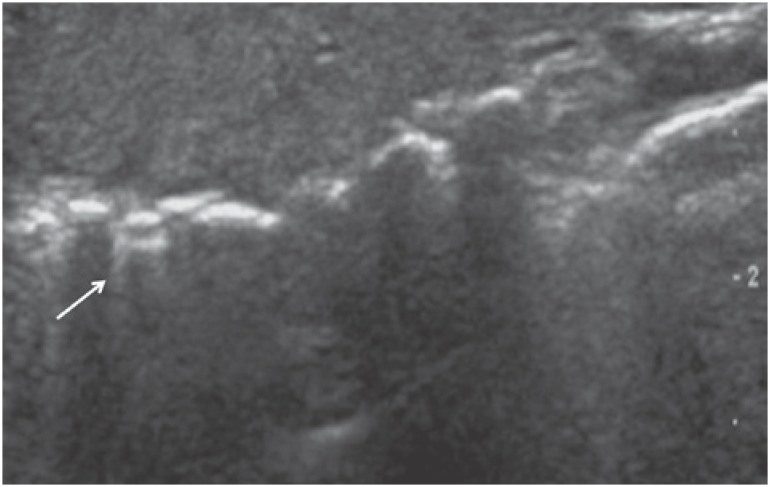
E lines. Subcutaneous emphysema generates lines (E lines) which arise from the superficial gas, not from the pleural line

“E” stands for subcutaneous emphysema. E lines are vertical lines seen when there is gas trapped in the subcutaneous space. These lines do not arise from the pleural line, but from the subcutaneous tissue; given the gas does not move, they are not synchronous with respiratory movements (Figure 5). E lines are well-defined and also erase A lines, and may therefore be mistaken for true B lines.

#### Z lines

**Figure 6:**
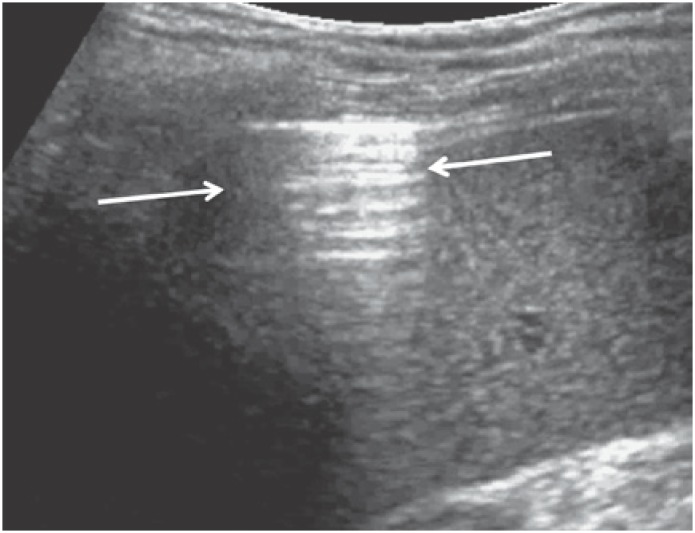
Z lines are common artifacts seen in more than 80% of the population. These bundle-shaped lines arise from the pleural line (arrows), do not erase A lines and do not move in synchrony with respiratory movements

Z lines (Figure 6) are common artifacts seen in more than 80% of the population and may be mistaken for coalescent B lines described above. Z lines are vertical, bundle-like shaped lines arising from the pleural line; however, they are ill-defined, do not erase A lines and are not perfectly synchronous with respiratory movements.^(3)^

## LUNG CONSOLIDATION

Lung consolidation is the increase in lung density, often in response to infectious conditions; the lung parenchyma appears similar in density to the liver due to replacement of normal lung air content by denser material. The condition is amenable to sonographic characterization provided there is no normal lung parenchyma between the pleural line and the consolidated lung (*i.e.*, sonographic visualization is limited to peripheral lung consolidation).

Consolidated lung appears tissue dense in sonography. Consolidation areas may be small, peripheral or large, highlighting permeated hyperechoic foci that are air bronchograms.^(3,5)^

Flow mapping using color or power Doppler may be helpful to differentiate between lung consolidation and atelectasis.^(6)^

Ultrasonography is also indicated for lung recruitment estimation in intensive care unit patients requiring assisted ventilation.^(7)^

### Pneumothorax

**Figure 7:**
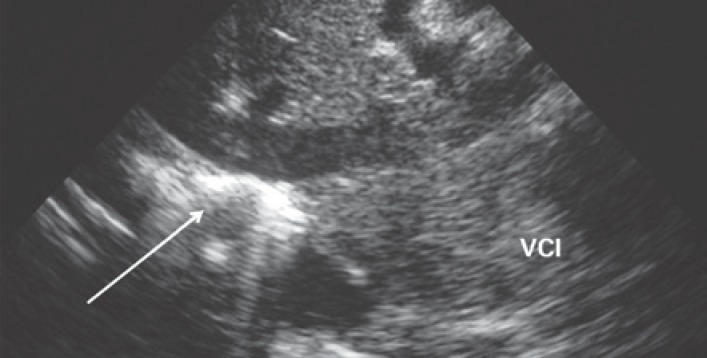
Pneumothorax. Lung sliding is absent (dynamic sonography); therefore, the pleural line (arrow) does not move as the patient breathes in and out VCI: inferior vena cava.

Pneumothorax diagnosis can be based on three straightforward criteria:^(8–11)^ (1) gas trapped in the pleural space obscures visualization of the underlying pleural line, so that no pleural sliding is seen. The presence of hemythorax and the absence of the lung sliding sign suggest pneumothorax (Figure 7); (2) search for B lines. B lines representing interlobular septa arise from the pleural line and can only be seen if this line is visible. The pleural line is not visible in pneumothorax; therefore, the presence of B line rules out pneumothorax;^(2)^ (3) identification of the “lung point” indicating expansion of the aerated lung into the pneumothorax.

Sonography is thought to have high accuracy in diagnosing pneumothorax and may outperform radiography in detecting small localized pneumothorax.^(3)^

## ULTRASOUND SCANNING PROTOCOLS

Some of the current lung ultrasound protocols indicated for management of patients with acute conditions are listed below.

– Blue Protocol^(1)^ (Bedside Lung Ultrassonography in Emergency): emergency protocol for immediate diagnosis of acute respiratory failure. This protocol describes specific sonographic findings associated with major conditions, such as pneumonia, congestive heart failure, chronic obstructive pulmonary disease, asthma, pulmonary embolism and pneumothorax, with more than 90% diagnostic accuracy.
– FALLS (Fluid Administration Limited by Lung Sonography) protocol:^(11)^ this protocol was designed to sequentially rule out differential diagnose, such as cardiogenic and hypovolemic shock, and allows for early diagnosis of septic shock.
– C.A.U.S.E. (Cardiac Arrest Ultrasound Exam) protocol: this protocol definitely normalizes the use of ultrasonography in cardiac arrest management. The undisputable indication of the technique in such cases and its value in detecting electromechanical dissociation are emphasized.

The ability to recognize and interpret B1 lines (inter and intralobular septal thickening) and B2 coalescent lines (ground glass) contributes to improved diagnostic accuracy. Description of major echogram patterns are given below:^(1,3,9–11)^

**Figure 8:**
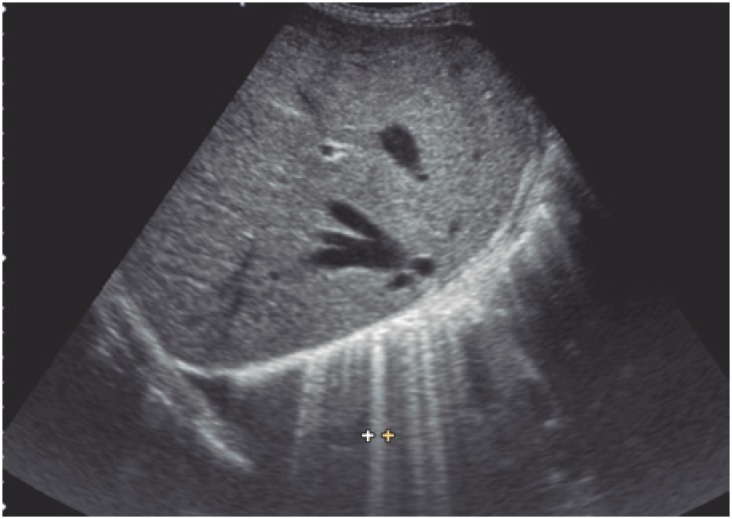
B1 lines (arrows) typical of hemodynamic edema

**Figure 9:**
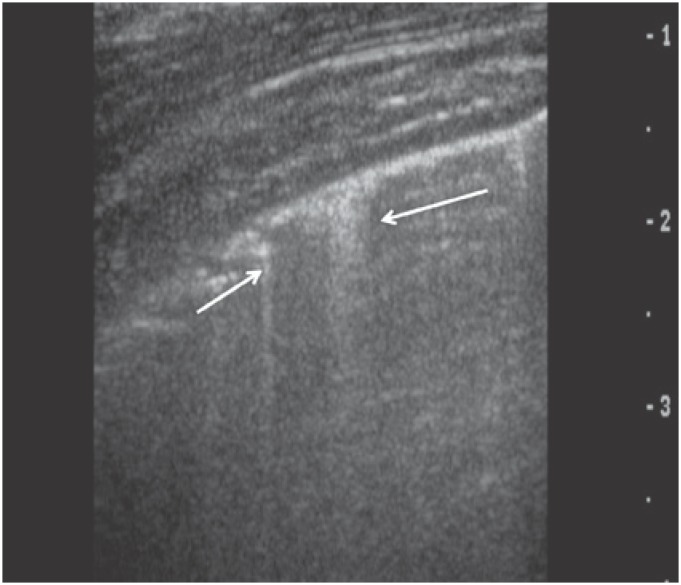
Coalescent B2 lines (ground glass pattern)

– Hemodynamic edema: evenly spaced (approximately 7.0mm apart; distance equal to secondary pulmonary lobe diameter) B1 lines (Figure 8). The B lines represent thickened interlobular septa, just like in high-resolution lung CT. The B2 lines (Figure 9) are also present with this condition and represent the ground glass pattern.
– Interstitial pneumonia: thickened interlobular and intralobular septa generate multiple, unevenly spaced B lines. Coalescent B lines (ground glass pattern) and decreased lung sliding motion may eventually be seen.
– Alveolar pneumonia: represented by lung consolidation signs described above. Even minute (millimetric) consolidations can be detected using ultrasonography, provided lesions are juxtapleural (peripheral).

## DISCUSSION

Combined chest sonography and radiography are often sufficient for diagnosis and management of pulmonary conditions in hospitalized patients, particularly in emergency room, intensive care or stepdown units.^(10,11)^

Sound knowledge of these techniques by radiologists, emergency and intensive care physicians may be crucial for proper management of several cases, reducing time to implement therapy, as compared to time required for high-resolution lung CT. Risks involved in patient transportation to the CT unit and the high financial and operational costs of CT should also be taken into account.^(1–5)^
